# People's experiences living with peripheral neuropathy: a qualitative study

**DOI:** 10.3389/fpain.2023.1162405

**Published:** 2023-06-28

**Authors:** Maryam Alkandari, Amelia Hollywood

**Affiliations:** School of Pharmacy, University of Reading, Reading, United Kingdom

**Keywords:** patient experience, self-efficacy, peripheral neuropathic pain, peripheral neuropathy, quality of life, qualitative research

## Abstract

**Introduction:**

Peripheral neuropathy is a neurological disorder characterised by pain, numbness, or tingling due to nerve damage. Peripheral neuropathy is one of the main health issues in Kuwait and is a rising concern which affects a large proportion of the population, therefore the lived experience needs to be explored to identify areas for improvement in care. This qualitative study explored the experiences of people living with peripheral neuropathy in Kuwait.

**Methods:**

Semi-structured interviews were conducted with 25 participants recruited from the Neurology Outpatient Clinic of the Ibn Sina Hospital in Kuwait. The interview questions explored their experiences and understanding of pain along with the impact on their daily life. The interviews were audio recorded, transcribed and translated into English then coded using NVivo 12. Thematic analysis was conducted to identify patterns and themes in the data.

**Results:**

Three major themes were identified including treatment beliefs (perceived effectiveness of treatment and seeking alternative treatments), the barriers to pain management (medication side effects, relationships with healthcare professionals and lack of information and access to healthcare), and the impact on quality of life (impact on work and social, physical, and psychological consequences). Self-efficacy was a key construct and over-arching theme that was discussed in all aspects, which finds reflection in the protection motivation theory.

**Discussion:**

This paper presents the experiences of people living with peripheral neuropathy and highlights there is scope for improvement of current treatments in Kuwait. Self-management strategies are recommended alongside prescribed medication and healthcare professionals are encouraged to use a patient-centered approach. More importantly, information and support on the condition to promote coping strategies and self-efficacy should be adopted to improve quality of life. These findings can be implemented locally and globally to improve the quality of care provided to people living with peripheral neuropathy.

## 1. Introduction

Peripheral neuropathy (PN) is a condition that involves damage to the peripheral nervous system (1). It is often disturbing, disabling, and can even be fatal. Peripheral neuropathy refers to pain affecting the peripheral nervous system caused by injury or disease. Several disorders are linked to peripheral neuropathy, including post-herpetic neuralgia, Human Immune Deficiency Virus (HIV), diabetic neuropathy, ischemic neuropathy and cranial neuralgias (e.g., trigeminal neuralgia). Moreover, peripheral neuropathy may also be from iatrogenic causes such as chemotherapy and limb amputation, or from infection such as Guillain-Barré syndrome (GBS). Nerve trauma and entrapment, such as in cervical radiculopathy and carpal tunnel syndrome, can also cause peripheral neuropathy (1). Peripheral neuropathy may also be caused by other factors, including diabetes and medication such as cisplatin and taxanes (2, 3). Symptoms of peripheral neuropathy include pain, numbness, tingling, a burning sensation, muscle cramps, muscular weakness/palsies, loss of vibration sensation, loss of perception of position, gait instability, and falls (3). Peripheral neuropathy exists worldwide and affects more than 20 million individuals in the United States alone, thus it is rapidly becoming a public health concern (3). Peripheral neuropathy is more common among the elderly compared to younger individuals (4). According to one study, the prevalence of peripheral neuropathy among the general population is 1%, but increases to approximately 7% in adults over 65 years old (5). In Western countries, such as the United Kingdom (UK), pain presents in approximately two-thirds of people living with peripheral neuropathy (6, 7). Peripheral neuropathy has been shown to cause a marked reduction in physical, social and emotional functioning (8). One study reported that diabetic patients with peripheral neuropathy had a significantly reduced quality of life compared to diabetic patients without peripheral neuropathy (9). Furthermore, another study showed that peripheral neuropathy had a negative effect on the sleep and work life of these individuals (10).

There has recently been a growing interest in peripheral neuropathy from the individual's perspective. Peripheral neuropathy has the potential to significantly disrupt the lives of people living with the condition, specifically the elderly. Vulnerability is found more in the elderly, who experience a loss of confidence in their ability to perform daily activities after experiencing the negative effects of peripheral neuropathy (11). A better understanding of the perspectives of people living with peripheral neuropathy would need to include an exploration of the support these people receive from their family and friends.

Peripheral neuropathy is present in 54% of people living with diabetes in Kuwait (12). A significant rise in the incidence of diabetes mellitus is seen with age (13). Peripheral neuropathy from infections and inflammatory conditions can also occur. The most common infection that causes peripheral neuropathy is HIV, but it is also seen in patients infected with herpes simplex virus, hepatitis C, the cytomegalovirus, West Nile and Epstein-Barr virus and the rabies virus. Bacterial infections can also cause peripheral neuropathies, which include Borrellia burgdoferi, diphtheria, Campylobacter jejuni, Mycobacterium tuberculosis, Mycobacterium leprae, Brucella, and Clostridium botulinum (14). Studies on people living with peripheral neuropathy in Kuwait have reported that pain negatively affects mood, sleep, relationships, and functional capacity (15).

The diagnosis and treatment of peripheral neuropathy is vital in being linked to both the diagnosis and management of treatment of the underlying condition. Peripheral neuropathy in Kuwait is diagnosed through a combination of techniques that include a neurological examination (full neurovascular examination and nerve conduction study) and a detailed medical history (physical examination and investigations including a blood test for HbA1c level). A number of pharmacological treatments are used in the management of peripheral neuropathy in Kuwait. The most commonly used medications are ketoprofen, etoricoxib, ibuprofen, acetaminophen, alpha-lipoic acid, vitamin b complex and pregabalin. Other medication include tricyclic antidepressants (amitriptyline), selective serotonin and norepinephrine reuptake inhibitor (duloxetine), and opioids (tramadol). Furthermore it is worth noting that topical applications such as capsaicin cream, are not available in Kuwait.

Previous research has mapped the experience of people living with peripheral neuropathy through the healthcare system in Kuwait (15). Although the management guidelines for peripheral neuropathy are similar in Kuwait to those in Western countries, such as the UK, there is no integrated referral system for consistent and connected health care. Moreover, patient education on self-management and coping strategies is lacking in Kuwait (15). Multidisciplinary care for people living with peripheral neuropathy is encouraged in Western countries, with many receiving a wide range of additional support (16). Part of this multidisciplinary care includes the promotion of self-efficacy, which is the confidence in one's ability to perform a particular behaviour (17, 18). Self-efficacy has been shown to be useful in promoting behaviour change in relation to pain management, such as in foot self-care in the elderly people living with diabetes in Malaysia (19).

A range of studies were conducted in the Western world examining the experiences of people living with peripheral neuropathy at various levels including the psychological (thinking optimistically) and emotional (such as conversing with family or friends) and physical (including basic exercise and performing simple tasks) (11, 20–24). The main objective of the current study was to explore the lived experience from a non-western perspective, in order to provide global insight into this health condition and expand on what it already known about the healthcare pathway in Kuwait. With growing numbers of people living with peripheral neuropathy in Kuwait it is important to identify and address any unmet needs. The aim of the study was to examine the experience of people living with peripheral neuropathy in Kuwait.

## 2. Materials and methods

### 2.1. Design

This study used an exploratory qualitative study to explore the experiences of people living with peripheral neuropathy in Kuwait. A semi-structured interview schedule was used, which contained open-ended questions, with the aim of examining the impact of the condition on the participants. A previous paper utilised the same data set to outline the pathway employed in the management of peripheral neuropathy in Kuwait (15). That study used a process map to highlight the patient journey from the perspective of clinical management and created a schematic map of the process. The current study employed thematic analysis, which moves beyond just reporting the data and instead interprets the data to provide in-depth insight into peoples' experiences.

### 2.2. Setting

The setting for this study was the Neurology Outpatient Clinic of the Ibn Sina Hospital. This clinic is one of the main clinics that neurology patients are referred to in Kuwait, when presenting with peripheral neuropathy. Ethical approval was obtained for this study from the University of Reading Ethics Committee, as well as from the Standing Committee for Health and Medical Research Coordination in the Ministry of Health in Kuwait. The semi-structured interview schedule was developed following an extensive literature review.

### 2.3. Recruitment

Permission was obtained from the Dean of Medicine at the Ibn Sina Hospital to conduct the study on the premises and for the neurologists attached to the Neurology Outpatient Clinic to identify eligible participants. The inclusion criteria consisted of participants being a resident of Kuwait, able to speak either Arabic or English, over 18 years old, diagnosed with peripheral neuropathy (any duration) and referred to a specialist.

Potential participants identified by the neurologists were provided with an invitation pack by the nursing staff in the clinic. This invitation pack consisted of an invitation letter, patient information sheet and consent form. Potential participants were asked to contact the study investigators if they were interested in the study. Completed consent forms were obtained from each participant and securely stored. Interviews were conducted in a private room at the Ibn Sina Hospital.

### 2.4. Procedure

The nursing staff approached 95 potential participants, of which 27 contacted the study investigators. Through screening two potential participants were excluded: one was under 18 years old and the other had a neurological condition that was not peripheral neuropathy. Twenty-five participants were selected for the study (26% recruitment rate) and consent was obtained. Demographic and medical information was collected through a review of the participants' hospital records prior to each interview, with prior informed consent obtained. Interviews were conducted in either Arabic or English, depending on the language preference of the participant.

A semi-structured interview schedule was used, with the aim of examining the impact of the condition on the participants. The interview schedule contained a range of open-ended questions including; Tell me about your pain? How does it make you feel? What did you know about peripheral neuropathy before you were diagnosed with it? Has your experience of symptoms changed over time? Do you explain these types of symptoms to other people? How effective do you feel medical treatment has been on treating your pain? Do you have any particular ways of coping? Each interview lasted between 45 min and 1 h (mean 51 min). Interviews were audio recorded and subsequently transcribed verbatim. Interviews conducted in Arabic were transcribed in Arabic then translated into English. Interviews conducted in English were transcribed directly. The transcriptions and translations were checked for accuracy with a sample of transcripts submitted for review. Four transcripts reviewed by a bilingual lecturer (proficient in both Arabic and English) and two by a similarly bilingual physician.

### 2.5. Data analysis

The verbatim transcripts were transferred to NVivo 12 software (QSR International, Melbourne) (25) which is a program used to organise data and facilitate analysis. The analysis method used in this study was thematic analysis, which identifies recurring patterns in the data, then analyses and interprets these to develop themes and draw conclusions (26). This methodology was employed because it provides a framework to deepen the understanding of peoples' experiences of living with peripheral neuropathy and offers an interpretation through the extracted themes. Thematic analysis was chosen as it involves more than just reporting the data and instead involves interpretation of the data in relation to the research question and therefore helps to gain an in-depth insight into the experiences of people living with peripheral neuropathy in Kuwait.

The analysis involved initially reviewing hard copies of the transcripts and annotating, to highlight text of interest. Following familiarisation, the transcripts were transferred to NVivo for the data to be manually coded with a combination of text search and coding queries executed to extract preliminary nodes and themes. The latter was performed by grouping codes to identify distinct themes. The suitability of each theme was then reviewed by integrating the data to generate a final list of themes with illustrative quotes. An example of a preliminary node and theme is the side effect of prescribed medication (such as pregabalin, brand name Lyrica) hindering the management of pain from peripheral neuropathy. This theme, which is a barrier to pain management, was then interpreted to highlight other barriers. The selected themes were then discussed with peers who were experts on neuropathic pain and in particular peripheral neuropathy. Table 1 depicts an example of how the initial codes were constructed from the quotes and how the themes were interpreted from the data.

**Table 1:** An example of the transformation of data through thematic analysis.

Quotes	Codes	Theme
“*I get nervous and feel that everything angers me, and my body as a whole hurts me*” *(Female, 56 years old, PN- 30 years)*. “*this pain affects me and makes me always nervous*” *(Male, 47 years old, PN- 3 years)*. “*I feel numbness in my hand … I was crying, crying and getting nervous*” *(Female, 36 years old, PN- 10 years).*	Emotional and Psychological effects	**Impact on quality of life**
“*… it affects me as I have to do everything slowly or sit down because when I feel the pain, I may drop the child or something like that …. of my friends told me that lately, I rapidly and suddenly get angry …*” *(Female, 62 years old, PN- 17 years)*. “*… before having these pains, I had several friends … As for now … social activities are no longer more*” *(Female, 56 years old, PN- 7 years)*.	Environmental and Social (Family, friends)	**Impact on quality of life**
“*I work with students with special needs who need normal person to help them and I can’t continue as most of the time I was standing, bending, walking and moving …*” *(Female, 62 years old, PN- 17 years)*. “*pain of my foot increases my nervousness and co-workers feel that there is something inside me boiling, which causes me to explode by talking to the staff and colleagues*” *(Female, 45 years old, PN- 9 years)*.	Tension and Embarrassment at Work	**Impact on quality of life**

## 3. Results

The aim of this study was to explore the experiences of people living with peripheral neuropathy in Kuwait. The majority of participants in this study were Kuwaiti, female, with an average age of 55 years and lived with peripheral neuropathy for 13.76 years. Participants mainly experienced neuromuscular symptoms; including physical pain in the feet, hands, extremities, back, leg and knees, as well as describing numbness and tingling. Table 2 includes the characteristics of participants included in the study.

**Table 2:** Characteristics of the participants included in the study.

Characteristics		Observations (*n* = 25)
Nationality	Kuwaiti	*n* = 20 (80%)
	Non-Kuwaiti	*n* = 5 (20%)
Sex	Male	*n* = 12 (48%)
	Female	*n* = 13 (52%)
Age (years)	Mean (SD)	55 (10)
	Range	35–82
Comorbidities	Type 2 diabetes mellitus	*n* = 16 (64%)
	Hypertension	*n* = 10 (40%)
	Dyslipidaemia	*n* = 8 (32%)
Primary cause of peripheral neuropathy	Type 2 diabetes mellitus	*n* = 16 (64%)
	Guillain-Barré syndrome	*n* = 1 (4%)
	Carpal tunnel syndrome	*n* = 2 (8%)
	Cytomegalovirus	*n* = 1 (4%)
	Post-herpetic neuralgia	*n* = 2 (8%)
	Acute injury	*n* = 1 (4%)
	Limb amputation	*n* = 2 (8%)
Duration of peripheral neuropathy (years)	Mean (SD)	13.76 (7.40)
	Range	3–30
Over-the-counter treatments	Nonsteroidal anti-inflammatory drugs: ibuprofen (Ibuprofen) 200–400 mg tablets	*n* = 10 (40%)
	Analgesics: acetaminophen (Panadol) 500 mg tablets	*n* = 5 (20%)
	Topical Analgesics: ketoprofen (Fastum 2.5%) gel	*n* = 1 (4%)
	Capsaicin cream	*n* = 1 (4%)

**Figure 1:**
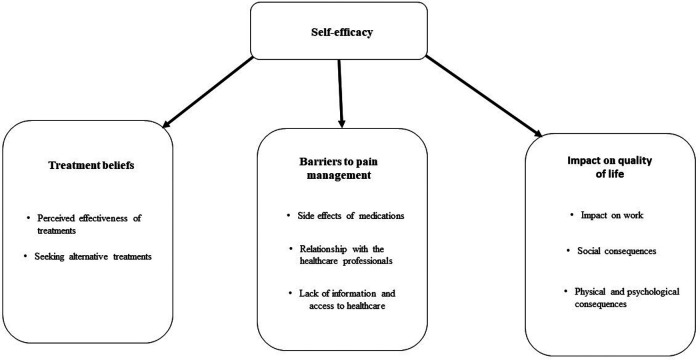
An illustration of the themes that were identified from people living with peripheral neuropathy.

The analysis identified three major themes: treatment beliefs, barriers to pain management and the impact on quality of life. The overarching theme which was present throughout was self-efficacy. The themes identified through the interviews are illustrated in Figure 1.

### 3.1. Treatment beliefs

Participants noted the importance of pain management and its role in improving their daily functioning. Different strategies were used to counteract pain including over-the-counter medication and complementary and alternative treatments.

#### 3.1.1. Perceived effectiveness of treatments

Prescribed medication, such as Lyrica and over-the-counter pain relief, such as acetaminophen (Panadol) and ibuprofen tablets, were reported to be used by most participants. They identified over-the-counter pain relief to be most useful in tackling both physical and psychological symptoms.

*I would not be able to sleep at night. I would be in a lot of pain and would have to wake up and take some Panadol. Thank God we have painkillers. (Female, 56 years old, PN- 30 years)*

Participants also reported the use of topical analgesics, such as ketoprofen (Fastum) 2.5% gel and capsaicin cream, to provide pain relief. Although these products are of limited clinical benefit in peripheral neuropathy, some participants still reported feeling relief by incorporating them into their treatment plans. One participant mentioned that she was very resistant to changing her use of the topical analgesic, capsaicin cream, even though she had to to procure this during travel abroad, as capsaicin cream was not available locally in Kuwait.

*The pain lessens when I use the capsaicin cream for my feet, thank God. I can stand up again when I use the cream. Frankly, I have been using it for a long time. This is my cream. I never changed it, although many women tell me that I have to change my pain medication, but I refuse it. I do not change my medication and will keep it until I die. (Female, 82 years old, PN- 20 years)*

#### 3.1.2. Seeking alternative treatments

Some participants sought out complementary and alternative therapies independently, such as Korean therapy-sujok, Chinese therapy-acupuncture and massage, to manage their pain. These participants did not consult their healthcare professional before starting the treatments. Information was obtained from shared experiences by colleagues, family, and friends. In particular, Korean therapy-sujok was mentioned as providing remarkable immediate pain relief.

*I practice the Korean therapy- sujok, which relieves the pain immediately within two minutes and its effects are immediately felt. As for painkillers, if you take them, they will need 10 to 20 min, but this sujok therapy takes only seconds. This is really unbelievable. (Male, 43 years old, PN- 4 years)*

In addition, several participants mentioned trying massage and acupuncture to alleviate their pain. The use of these alternative treatments allowed participants to feel in control of their symptoms.

*I massage my fingers, close my hands and move my hand in warm water for some time. To reduce my pain, I regularly go for acupuncture sessions when I'm free. (Male, 62 years old, PN- 10 years)*

As a natural alternative to medication, herbal therapy (for example, ginseng tablets and cinnamon tea) was also used by a number of participants. Although many did not fully endorse the efficacy of herbal therapy initially, they continued with it as they believed it was unlikely to do any harm. Participants also felt that herbal therapy was worth pursuing without prior discussion with their healthcare professionals, who did not pay much attention to alternative treatments.

*Well, whenever I feel pains, I take two tablets of ginseng per day, in the morning at breakfast and in the evening at dinner. It's a herbal treatment for my legs to reduce the pain and I decided to take it without asking my doctor and he never asked me about it. (Female, 56 years old, PN- 7 years)*

### 3.2. Barriers to pain management

Participants reported several barriers to pain management when discussing the experiences of living with peripheral neuropathy. The main barriers discussed included the side effects of medication, relationships with healthcare professionals and a lack of information and access to healthcare.

#### 3.2.1. Side effects of medication

Participants reported that the side effects of prescribed medication had a significant impact on their pain management. A key factor discussed by a number of participants for discontinuing the use of Lyrica tablets was the side effects. Participants held negative views of the medication they were prescribed and expressed concerns about continuing the entire course of their medication due to the side effects.

*Due to the countless side effects from Lyrica, including weight gain, I no longer take the dose that the doctor prescribed for me. (Female, 45 years old, PN- 9 years)*

#### 3.2.2. Relationship with the healthcare professionals

Participants discussed how their healthcare professionals would focus on the medical management of the disease rather than highlighting the importance of behaviour change and coping with the illness. Participants reported that this approach led to emotional consequences such as fear, anger and stress, which meant they did not adhere to the medication.

*I am saddened by the doctors' apathy. This apathy and neglect led me to neglect myself. The stress coupled with the severe pain and neglect led to stop taking my own medication. I wish doctors considered our pains and feelings in order to examine us more carefully. (Female, 45 years old, PN- 9 years)*

Some participants reported that the limited duration of the consultation with their healthcare professional resulted in a perceived lack of empathy. This also led to a lack of information received by participants about their condition and its management, leaving them with uncertainty and feeling in a worse state than they were before the consultation.

*They should give enough time and not rush even if they have a second patient. They need to examine and listen to me as it should be. They also should give me more than just [medical] treatments. (Female, 36 years old, PN- 10 years)*

#### 3.2.3. Lack of information and access to healthcare

Several participants reported “suffering in silence” with their condition. These participants highlighted that they did not have access to useful information about peripheral neuropathy and its management, with some noting a lack of motivation to join a support group for their condition as the concept of the support groups is new in the Kuwaiti community.

*There are no such seminars that educate patients. The doctor also did not speak about such seminars and did not give me brochures. (Female, 82 years old, PN- 20 years)*

Many participants felt unable to manage their condition due to long waiting times in receiving pain relief. Participants preferred immediate treatment rather than waiting many months.

*As for the referrals, they are the same for the person who feels pain and the person who does not feel pain. For example, the doctors, give a medical appointment for three months, and if you miss your medical appointment, it is a big problem. By the time I get to the doctor I am tired and feel bad.(Female, 56 years old, PN- 30 years)*

### 3.3. Impact on quality of life

Participants noted that their quality of life was negatively affected by their condition. The physical and psychological symptoms of peripheral neuropathy impacted their work, emotional and social lives.

#### 3.3.1. Impact on work

The workplace was reported to be an unsupportive place by some. Mechanisms were not put in place to help participants manage their condition. A few participants recommended that receiving assistance from colleagues in completing certain tasks would help make the workplace more manageable.

*In fact, the nature of my work obliges me not to sit at the office, where I should go and come back because I am responsible for the whole section. If I finished work and sat down, I will begin to feel pain, forcing me to rise again and move to press on my feet, then I ask my friend at work to walk me to another division in order to move and press my feet because if I press them, the pain will decrease, but if I continued to sit, the pain will continue. (Female, 45 years old, PN- 9 years)*

#### 3.3.2. Social consequences

Some participants noted that the misunderstanding of their condition by other people resulted in social isolation and loneliness.

*Do you mean that I talk to anyone? No. Because of my pain, I now have a different routine from most men. Most men go from the house to the mosque to religious seminars to the Diwaniyah [men’s gathering] once a week where they talk about themselves. I do not like talking about myself and my pain, which is why I do not go to the Diwaniyah. To be honest with you, I do not want to go to the Diwaniyah anymore because of the pain. (Male, 62 years old, PN- 25 years)*

Several participants reported that strong emotions such as anger had become a part of their life. Some reported their family roles had become impaired and the happiness of their children was affected.

*I have become so nervous that I fight with the children a lot and I make a problem about something trivial. (Female, 45 years old, PN- 9 years)*

Most participants reported that their condition resulted in a loss of independence and weakened relationships with family and friends. Participants also felt that they were unable to fulfil their family roles.

*Sometimes I want to change my clothes and put on trousers. I cannot, so I ask my wife for help and here I begin thinking that I have reached such a degree that I cannot change my trousers. (Male, 60 years old, PN- 15 years)*

This situation was further worsened when participants felt embarrassed and were unable to openly discuss their condition with their own family members.

*I also do not consider it a subject of discussion even with my wife because she is the only one who helps me when feeling pain, particularly during traveling or walking together. (Male, 64 years old, PN- 18 years)*

#### 3.3.3. Physical and psychological consequences

Participants who were left uninformed about their condition reported stress and discomfort in not knowing whether their condition was improving or getting worse. A number of participants described experiencing fear that resulted in mood swings and emotional outbursts.

*I feel a sense of fear when I am at work. I am afraid of feeling pain while I am working. (Male, 62 years old, PN- 10 years)*

Other participants reported a lack of sleep and a sense of vulnerability due to physical symptoms such as tingling and stabbing.

*Due to diabetes I had severe tingling in the extremities of my hands and feet. Not only that, I also felt a kind of burning in my hands and feet. It was a kind of heat that led to sense the tingling in my hands and feet, particularly when sleeping. (Female, 60 years old, PN- 4 years)*

### 3.4. Over-arching theme of self-efficacy

The over-arching theme discerned throughout the data was self-efficacy, apparent in all subthemes. Self-efficacy refers to the way in which an individual believes in their ability to execute behaviours to achieve certain attainments (17). Self-efficacy was reflected in the first theme, which was treatment beliefs, in relation to dealing with the pain associated with peripheral neuropathy by developing resilience and exhibiting a positive attitude towards over-the-counter treatments.

*I also asked the doctor to refill my prescription of Panadol pills, which are the first thing I get refilled from the clinic. Later on, when I met the doctor, she told me that Panadol pills are excellent for me. Thank God! When I used it, I relaxed a lot. We must continue to fight the pain. (Female, 56 years old, PN- 30 years)*

The second theme, which was barriers to pain management, had an impact on the perceived control people experienced in relation to their condition. Some reported low self-efficacy in that they did not feel capable of coping with and managing their pain. A number of participants highlighted their interactions with healthcare professionals and the healthcare system impacted on their ability to control their pain. Participants described the lack of empathy from their healthcare professionals lead them to stop taking their medication, which was a barrier to pain management.

*I informed the doctor about the severe numbness and other pains. He said that he cannot do anything about it until the pain grows enough to do further tests. The fear of pain coupled with the doctor's neglect affected me and discouraged me from taking my medication. (Male, 43 years old, PN- 4 years)*

The third theme identified in this study was the impact of peripheral neuropathy on the quality of life of participants. Self-efficacy was apparent in this theme as participants reported feeling unable to complete tasks required as part of their working lives.

*I feel tired and exhausted. I can’t complete work, or I need to take a break and then go back to complete the work. (Male, 62 years old, PN- 10 years)*

Self-efficacy was a dominant construct throughout the participants account of their experience living with peripheral neuropathy in Kuwait. These data provide global insights into the experience of living with peripheral neuropathy from a new perspective.

## 4. Discussion

The findings of this study give insight into the experiences of people living with peripheral neuropathy in Kuwait, with a focus on coping and management strategies. Specifically, these included avoiding the prescribed medication (i.e., Lyrica), camaraderie in sharing experiences with others in a similar situation, and the use of alternative medicine (such as Sujok therapy and acupuncture). The first theme identified in the present study highlights the participants treatment beliefs and their perceptions of the effectiveness of treatments and alternative treatments that they explored. Patients were aware that pain management was a key factor in improving daily life. The “instant relief”, as well as physical and psychological comfort described by participants in relation to the use of over-the-counter medications, supports current guidance used as standard practice in Europe. This guidance suggests self-management through the use of over-the-counter analgesics for localised neuropathic pain (27). A total reliance on over-the-counter pain medications should be noted with caution as a recent study regarding self-medication with over-the-counter analgesics reported that 40% of the sample showed substantial concern about the perceived need for pain medication and the perceived potential for harmful effects (28). The use of alternative therapies and massage to counteract their pain and symptoms was usually an autonomous undertaking, conducted without consulting with their healthcare professional. This should be viewed as a means of contributing actively to their individual treatment and of gaining a sense of control over their health condition. The suggestion that basic self-help or alternative treatments such as massage and acupuncture helped in alleviating pain and stress is also supported by previous research (29). Nevertheless the use of herbal medication, even if there are no apparent side-effects, should be viewed with caution and discussed with a healthcare provider in case of potential interactions.

The second theme identified was barriers to pain management which were attributed to the side effects of medications, their relationship with the healthcare professionals and a lack of information and access to healthcare. The fear of side effects has often been reported worldwide as a contributor to a lack of medication adherence (30–32). Relationships with healthcare professionals was noted as strained by the participants, with a need for longer consultations and empathy from doctors. Yu et al. (2022) found that the time physicians had spent on their health education, by keeping up to date on the effectiveness of treatments, had an important bearing on patient satisfaction (33). Paying attention to the emotional needs of patients (34, 35) and using empathic statements (36) has been demonstrated to result in better healthcare outcomes. The lack of information about their disease and its prognosis, experienced by our participants, needs to be tackled as access to health information is a key determinant of health. Governments have a legal responsibility under international human rights law to provide access to healthcare information to citizens and health workers (37). It is noted that longer waiting times are negatively associated with clinical provider scores of patient satisfaction and that every aspect of the patient experience, specifically confidence in the care provider and perceived quality of care, correlated negatively with longer waiting times (38). Moreover, in our study, many participants felt unable to manage their condition due to long waiting times in receiving pain relief.

The final theme from the thematic analysis examined the impact on quality of life. The sub-themes identified were the impact on work, along with social, physical and psychological consequences. Work environment was often reported as unsupportive, especially where mechanisms were not put in place to assist people living with peripheral neuropathy. The importance of workplace assessments as well as notifying employers and colleagues about the condition can help make the work environment a better place (39). Social isolation and loneliness were reported by many participants in the current study. Parallels were found between the findings of this study and those made by Aloisi et al. (40) where patients developed a fear of becoming weak or disabled and of their increasing dependency on family and friends (41). Closs et al. (20) suggested that health outcomes were primarily challenged by a reduction in the quality and/or number of personal relationships that the patient had, which was similar to those reported in this study. The physical and psychological consequences of living with peripheral neuropathy reported by our participants, such as lack of sleep, mood swings and anxiety, are coherent with the findings of the long-term effects of chemotherapy-induced peripheral neuropathy (42) and the mood disorders witnessed in neuropathic pain (43).

The results of this study highlighted an overarching theme of self-efficacy. Through the treatment beliefs, barriers to pain management and the impact on quality of life; self-efficacy was apparent. Previous research has suggested that self-efficacy partially mediates the association and changes in pain and disability (44). High self-efficacy has been associated with better functional outcomes, and variability in self-efficacy mediates the association between pain intensity and disability (45). This has been observed in various other pain conditions such as arthritis, headaches (46, 47), fibromyalgia (47, 48) and paediatric pain conditions (48, 49).

The themes identified in the current study find reflection in the protection motivation theory. This theory proposes that people protect themselves based on the perceived severity of a threatening event, the perceived probability of the occurrence or vulnerability, the efficacy of the recommended preventive behaviour (response-efficacy), and the perceived self- efficacy (50). These findings provided support for self-efficacy expectancy as a fourth component of the protection motivation theory: Self-efficacy has a strong impact on intentions and it is connected with two other components of the protection motivation theory (50). The theory stems from the concepts of threat-appraisal and coping-appraisal. Threat-appraisal incorporates severity and vulnerability along with the rewards of continuing the unhealthy behaviour or condition. Coping-appraisal includes the response-efficacy and self-efficacy (50). In this study, the severity of pain and symptoms of peripheral neuropathy were discussed in relation to the fear of disease progression. Vulnerability due to physical symptoms, lack of sleep and the lack of health information, along with a lack of proper treatment for peripheral neuropathy, led to a threat-appraisal. An adaptive coping-appraisal was demonstrated in this study through the response efficacy of effective treatment and self-efficacy from the use of over-the-counter products or turning to alternative therapy. With a high threat and coping appraisal, people living with peripheral neuropathy were motivated to seek better care.

### 4.1. Implications for research

The main strength of this study is that it gives global insights into the experience of living with peripheral neuropathy. Previous research mapped out the patient journey through the healthcare system in Kuwait (15). The current study provides an in-depth insight into the experiences and beliefs of people living with peripheral neuropathy in Kuwait, which was a gap in the literature and therefore offers a new perspective. The method employed, which was a semi-structured interview, enhanced the flexibility, sensitivity, reliability and depth of the data collected. Furthermore, the analysis enabled the identification of key themes surrounding the views of people living with peripheral neuropathy in Kuwait and their attitudes towards the treatment and practices of their healthcare professionals. The study also highlighted the relevance of the protection motivation theory in the field of peripheral neuropathy management which can provide the theoretical underpinnings for future research.

Some limitations of this study include the sample size, although this was adequate for the methodology used and data saturation was reached during analysis of the themes extracted. Furthermore, since the study was qualitative and employed a homogenous sample, its results cannot be generalised to everyone with peripheral neuropathy. Due to data saturation and no new themes emerging it could be suggested that the findings are representative and may reflect the experience of people living with peripheral neuropathy in Kuwait.

Further studies should explore novel methods to improve the lives of people living with peripheral neuropathy in Kuwait. Based on the findings of this study, potential areas of future research could include the impact of non-pharmacological treatments to improve physical and psychological well-being, this could involve a trial exploring the effectiveness of Sujok and acupuncture to improve pain management, which was a strategy used by participants in this study. Furthermore the impact of a multidisciplinary intervention, where medical doctors or specialists work in combination with complementary medicine to facilitate adaptive coping in individuals living with peripheral neuropathy, is also possible (52).

### 4.2. Implications for practice

The findings of this study offer several potential implications for clinical practice. Participants identified the benefits of alternative treatments, such as acupuncture, in their pain management. Therefore, healthcare professionals should offer advice and support to their patients by informing them of these potential self-management strategies. Furthermore, as participants identified concerns relating to the perceived effectiveness of prescribed medications, healthcare professionals should assist patients to understand how and when they should use over-the-counter medication and when to consult a physician. Recommendations should be made to promote effective self- management strategies and to prevent contraindications from some alternative treatments. All initiatives should be directed towards promoting self-efficacy in people living with peripheral neuropathy.

To achieve a patient-centred approach and to overcome potential barriers in pain management, healthcare professionals should practice compassion, trust and good communication. This is also important in pain management more broadly in Kuwait, which is focused on pharmacological management and lacks provision for psychological support including information and counselling sessions. Moreover, patient education on self-management and coping strategies for pain management is lacking in Kuwait (52). With respect to Kuwait, there is a need for an organised counselling network to aid patients and families, which could be supported by a 24 h hotline to respond to patient queries and concerns regarding side-effects, as other research has shown this has beneficial effects on patient outcomes (51). Furthermore, access to healthcare including appropriate treatments and medical appointments, especially the duration of the appointment, should be sufficient to address all aspects of patient care. Healthcare professionals should also consider a holistic approach to the consultation. A holistic approach takes into account all aspects of an individual's well-being that includes physical, emotional and mental perspectives. Several studies have demonstrated improvements in functional performance, quality of life and the alleviation of neuropathic symptoms following endurance training, balance training, and cognitive behavioural therapy. Therefore, healthcare professionals should consider offering behaviour change advice as part of the consultation or referring patients to appropriate therapists. This study highlights the importance of the workplace on the quality of life of people living with peripheral neuropathy. Workplace policies should promote the use of workplace assessments and managers should encourage a supportive environment to help people living with peripheral neuropathy remain in employment. Occupational therapists could be involved in multidisciplinary care to help people develop strategies to help them continue to perform activities that are important in their day-to-day life.

Patient-centred care is a pillar of most Western healthcare systems. Patient-centred care ensures the patient's voice is heard, that patients contribute to their own management plan and that they are made fully knowledgeable of all aspects of their care (52). Overall, the healthcare system in Kuwait needs to develop patient-centred care and welfare services further, including setting up peripheral neuropathy support groups where patients can connect with others. New services could be co-produced by patients and healthcare professional working together to develop a new approach that ensures the patient needs are met. Collaboration between people living with peripheral neuropathy and healthcare professionals can include the implementation of different practical approaches, such as educating people living with peripheral neuropathy about their condition, as well as supporting patient and family engagement with the new services. Healthcare professionals should provide people living with peripheral neuropathy with comprehensive information on existing treatments and on living with the condition. Such information should include the promotion of a healthier lifestyle, which can have a major impact on the physical and psychological well-being of people living with peripheral neuropathy. These approaches will involve people in their own care and improve overall patient outcomes.

## 5. Conclusion

This study provides new insights into the experiences of people living with peripheral neuropathy in Kuwait, with a focus on the coping mechanisms and management strategies. This study contributes to the literature by providing insights into the patient experience in a non-Western country and by identifying relevant themes to improve patient care. The treatment beliefs, barriers to pain management and impact on quality of life were key areas identified. The results reflect the extensive scope for improvement of current treatments and the management of peripheral neuropathy in Kuwait to improve people's quality of life. These can be achieved by individual behaviour change and by developing improved practice for healthcare professionals providing support to people living with peripheral neuropathy, to provide world-class healthcare.

## Data Availability

The datasets presented in this article are not readily available because the participants did not provide consent for their transcripts to be made publicly available.
